# The Teeth as an Index of Civilization

**Published:** 1893-12

**Authors:** R. B. Winder


AMERICAN JOURNAL

—OF—

DENTAL SCIENCE.

VOL. XXVII. BALTIMORE, DECEMBER, 1893. NO. 8.

ARTICLE I.

### THE TEETH AS AN INDEX OF CIVILIZATION.

BY DR. R. B. WINDER.

It is a noteworthy fact that among the barbaric tribes the jawbone is most highly developed, while the forehead is small. Take Australian bushmen, for example. They are supposed to represent as low a type of human beings as exist on the surface of the globe. They have powerful jaws, immense teeth, while the upper part of the head, or that part of the skull whose size indicates the possession of high mental faculties and reasoning power, is hardly more developed than that of the monkey tribe. The cranium suffers at the expense of the lower part of the face. It is a sure sign of civilization when the jaw of any nation is small and the teeth firm. The most beautiful faces of the highly-educated American women are those that are oval in form, or taper from the temple down to the chin. This was the case among the Grecian women, who were said to have been the most beautiful as well as the most accomplished of their day. The lowest types of the Caucasian race are those possessed of the strongest and squarest jawbones, and this can be seen among the prize fighters, 'toughs,' 'crooks' and criminals of the present day. Men of high literary attainments, culture and refinement seldom possess the square jaw of the pugilist, the coarse teeth and the broad expanse of chin—usually a characteristic of this class.

Take the mule, noted for its obstinacy, or the bulldog, noted for its ferociousness, and the truth of my statement can be readily verified. Among all savage races the powerful jaw and the strong, even teeth are attributes.

I once examined 1,000 skulls of the different Indian tribes, which formed a collection in Washington, and among this great number I found only 27 teeth decayed. The imperfect condition of these I attributed to some accident. The use of any organ or makeup of the human frame serves to strengthen the part, and this is the reason the teeth of people of civilized nations are not so strong nor firm as those of barbaric or semi-barbaric tribes, from the fact that the former have to perform the act of mastication less than the latter, since most of their food is well cooked and does not require such thorough chewing.

The teeth of the American people are said to be the worst of any in the world, and it is not due to the fact that they eat hot food, neglect them, nor anything of the kind. It is simply that Americans are made up of so many different nationalities that their teeth suffer in consequence. It is a strange thing that a child will usually inherit the form of jaw of the mother, with the teeth of the father, and this alone is the cause of so much irregularity, such unevenness in the ivories of the people of the states. There are said to be more irregular teeth in the city of St. Louis than in any other place the world over, and it has been justly attributed to the fact that so many Germans have there married the so called Anglo-Saxon American women. The teeth of the Teutonic race are large, while the jaws of the American women whom they marry are small. The consequence is there is not sufficient room for the large teeth inherited from the father to fit into the jaw inherited from the mother. They are crowded one into another.

It will probably be news to most persons to know that Baltimore has the distinction of having instituted the first dental college in the world, and having organized the degree of D. D. S., or Doctor of Dental Surgery, now used all over Europe. Yes, such is the case. For many years the Baltimore College of Dental Surgery was the only institution of its kind in the world. It was chartered in 1839 by an act of the Legislature of the State of Maryland, and is, therefore, in its 55th year. It was organized chiefly through the efforts of Dr. Chapin Harris, and on this account Baltimore may be said to be the cradle of dentistry and the dental profession.

George Washington had several sets of false teeth, two of which are owned in Baltimore. One of these sets is now on exhibition at the Columbian Exposition. The teeth in this were carved out of ivory and the plate was of gold. The other set, probably the first the immortal George ever had, is now in the possession of Edmund Law Rogers, who is a lineal descendant of Mrs. Washington. The plate of this set is lead and the workmanship is of a very crude and poorly-constructed nature.

